# Effects of Administration of Live or Inactivated Virulent *Rhodococccus equi* and Age on the Fecal Microbiome of Neonatal Foals

**DOI:** 10.1371/journal.pone.0066640

**Published:** 2013-06-13

**Authors:** Angela I. Bordin, Jan S. Suchodolski, Melissa E. Markel, Kaytee B. Weaver, Jörg M. Steiner, Scot E. Dowd, Suresh Pillai, Noah D. Cohen

**Affiliations:** 1 Equine Infectious Diseases Laboratory, Department of Large Animal Clinical Sciences, College of Veterinary Medicine and Biomedical Sciences, Texas A&M University, College Station, Texas, United States of America; 2 Gastrointestinal Laboratory, Small Animal Clinical Sciences, College of Veterinary Medicine and Biomedical Sciences, Texas A&M University, College Station, Texas, United States of America; 3 Molecular Research DNA Laboratory, Shallowater, Texas, United States of America; 4 National Center for Electron Beam Research and Department of Poultry Science, Texas A&M University, College Station, Texas, United States of America; Northeast Agricultural University, China

## Abstract

**Background:**

*Rhodococcus equi* is an important pathogen of foals. Enteral administration of live, virulent *R. equi* during early life has been documented to protect against subsequent intrabronchial challenge with *R. equi*, indicating that enteral mucosal immunization may be protective. Evidence exists that mucosal immune responses develop against both live and inactivated micro-organisms. The extent to which live or inactivated *R. equi* might alter the intestinal microbiome of foals is unknown. This is an important question because the intestinal microbiome of neonates of other species is known to change over time and to influence host development. To our knowledge, changes in the intestinal microbiome of foals during early life have not been reported. Thus, the purpose of this study was to determine whether age (during the first month of life) or administration of either live virulent *R. equi* (at a dose reported to protect foals against subsequent intrabronchial challenge, viz., 1×10^10^ colony forming units [CFU]) or inactivated virulent *R. equi* (at higher doses, viz., 2×10^10^ and 1×10^11^ [CFU]) altered the fecal microbiome of foals.

**Methodology/Principal Findings:**

Fecal swab samples from 42 healthy foals after vaccination with low-dose inactivated *R. equi* (n = 9), high-dose inactivated *R. equi* (n = 10), live *R. equi* (n = 6), control with cholera toxin B (CTB, n = 9), and control without CTB (n = 8) were evaluated by 454-pyrosequencing of the 16S rRNA gene and by qPCR. No impact of treatment was observed among vaccinated foals; however, marked and significant differences in microbial communities and diversity were observed between foals at 30 days of age relative to 2 days of age.

**Conclusions:**

The results suggest age-related changes in the fecal microbial population of healthy foals do occur, however, mucosal vaccination does not result in major changes of the fecal microbiome in foals.

## Introduction

*Rhodococcus equi* is a facultative intracellular pathogen that primarily infects macrophages [1]. Although human beings may be infected (primarily those who are immunocompromised by HIV infection or immunosuppressive treatments), *R. equi* is most commonly recognized clinically as a leading cause of severe pneumonia in foals [1]–[4]. The disease occurs among foals worldwide [1]–[4]. Isolates that are virulent in foals bear a plasmid that encodes for a pathogenicity island, which includes the gene for the virulence-associated protein A (vapA); vapA is necessary but not sufficient to cause disease [5], [6].

Despite the global importance of the disease, an effective vaccine is lacking for control and prevention of *R. equi* pneumonia in foals. The lack of an effective vaccine is likely attributable to the complexity of immunity to *R. equi* [7]–[9], and the finding that foals appear to be infected very early in life [10], [11], when immune responses are naïve or deficient. It is generally accepted that a vaccine must be able to provide foals with protection against infection with *R. equi* during early life [5].

To date, the only vaccination strategy that has been demonstrated repeatedly to be effective for protecting against experimental intrabronchial challenge with virulent *R. equi* has been oral administration of live, virulent *R. equi* [12]–[14]. Protection against respiratory pathogens induced by oral vaccination also has been documented in mice [15]–[17], and evidence exists that bacillus Calmette-Guerin (BCG) administered orally is protective against tuberculosis in people and animals [18]–[20]. Moreover, inactivated bacteria and viruses also can elicit protective immune responses against systemic infections, including those of the respiratory tract [21]–[24]. Despite the success of oral administration of live organisms to protect foals against experimental challenge, very limited information is available regarding immune and other biological responses to the enteral route of vaccination.

One issue of importance with regard to enteral vaccination with live organisms is the impact of enteral administration of bacteria on the intestinal microbiome. This question might be particularly important for neonates. Although the microbiome of foals has not been systematically evaluated, evidence exists in other species, including humans, that the intestinal microbiome of neonates develops with age [25]–[27], and is linked to the functional development of the gut and gut immunity [25]–[29]. Thus, the purpose of the study reported here was to determine whether age-related changes in the microbiome occur in foals and whether age-associated changes are impacted by administration of either live virulent *R. equi* at a dose documented to protect foals against experimental challenge or 2 doses of inactivated virulent *R. equi* higher than the dose of live *R. equi*.

## Materials and Methods

### Ethics statement

All procedures for this study, including collection of rectal swab samples and enteral treatments/vaccinations, were reviewed and approved by the Texas A&M University Institutional Animal Care and Use Committee (protocol number AUP # 2011-124) and the Texas A&M University Institutional Biosafety Committee (permit number 20110183-Cohen). The foals used in this study are owned by Texas A&M University, and permission for their use was provided in compliance with the Institutional Animal Care and Use Committee procedures.

### Animals and housing

Forty-two healthy Quarter Horse foals were used for this study. All foals were born healthy and had age-appropriate results of complete blood count (CBC) on day 2 of life, and adequate transfer of passive immunity as assessed by a commercially-available qualitative immunoassay for serum concentration of total IgG (SNAP Foal IgG test; IDEXX, Inc., Westbrook, ME). The foals were assigned into 1 of 5 experimental groups prior to birth (please see section on Vaccine Preparation and Treatment Groups below). All foals were monitored daily by Texas A&M University Horse Center staff for clinical signs of disease, and inspected at least twice weekly by a veterinarian for clinical signs of disease. All foals remained free of clinical signs of disease and in good health throughout the study.

### Mare Diet

The respective dams were fed 6.4 kg per horse per day of a 13% horse pellet (crude protein: 13.5%; crude fat: 4.5%; crude fiber: 10%). Also, the foals and their mares were allowed free access to coastal Bermuda grass hay, plus grazing of pastures at the Texas A&M University Horse Center where the mares were maintained.

### Vaccine Preparation and Treatment Groups

*Rhodococcus equi* strain EIDL 5-331, obtained from a Texas foal confirmed to have *R. equi* pneumonia, was used to prepare live and inactivated vaccines used for this project. Physiological saline (NaCl 0.9%) was used as a diluent to achieve the specified concentration of all vaccine preparations, as well as for the negative control. The vaccine was produced by inoculating blood agar plates with 1 colony forming unit (CFU) of *R. equi* strain 5-331 and incubating at 37°C for 48 hours. One colony from this pure culture was selected and used to inoculate 1,000 ml of brain heart infusion (BHI, Bacto™Brain Heart Infusion, BD Diagnostic Systems, Sparks, MD) broth. The flask with inoculated broth was placed on an orbital shaker (VWR OS-500, VWR, Radnor, PA) at 200 rpm for 24h at 37°C to allow bacterial growth. Isolates were repeatedly tested by PCR for the vapA gene to confirm that the isolates were virulent [30]. The bacterial culture was inactivated by electron-beam irradiation (irradiation dose between 3.5 and 5 kGy). After inactivation, the irradiated bacterial cells were plated out on BHI agar plates and incubated for 2 weeks at 37°C to confirm inactivation.

The number of foals in each group was determined *a priori*, and foals were assigned randomly to each of the groups. The study groups were as follows: 1) low-dose inactivated virulent *R. equi* group (n = 9), receiving 2×10^10^ CFUs of inactivated *R. equi* combined with 100 µg of cholera toxin subunit B (CTB, List Biological Laboratories, Campbell, CA) as a mucosal adjuvant, diluted in 100 ml of saline administered via nasogastric intubation; 2) high-dose inactivated virulent *R. equi* group (n = 10), receiving 1×10^11^ CFUs of inactivated *R. equi* with 100 µg of CTB diluted in 100 ml of saline via nasogastric intubation; 3) live virulent *R. equi* group (n = 6), receiving 1×10^10^ CFUs of live *R. equi* diluted in 100 ml of saline administered via nasogastric intubation; 4) control with CTB group (n = 9), receiving 100 µg of CTB diluted in 100 ml of saline via nasogastric intubation; and, 5) control without CTB group (n = 8), receiving 100 ml of saline via nasogastric intubation. Treatments (i.e., live bacteria, inactivated bacteria, and negative controls) were administered by nasogastric intubation to foals at 2, 9, 16, and 23 days of age.

### Fecal swabbing

Rectal swabs were collected by inserting a 16-inch, cotton-tipped swab that was pre-moistened with 3 ml of sterile saline approximately 2 to 3 inches into the rectum, and swabbing the rectal mucosa circumferentially by rotating the swab. Once the cotton swab was removed, the cotton tip was separated from the handle using scissors and the tip was placed inside the barrel of a 35-ml catheter-tip syringe; the syringe plunger was used to squeeze the liquid from the swab tip, and the liquid was collected into a sterile tube. Fecal swab samples were collected on days 2 and 30 of life from foals in all groups. For 2 foals in the control group without CTB, fecal swab samples were collected on days 2, 9, 16, 23, 30, and 56 following birth. All fecal solutions were frozen at -80°C until processed.

### Fecal DNA extraction

DNA was extracted by a bead-beating method using the ZR Fecal DNA MiniPrep Kit (Zymo Research Corporation) per the manufacturer’s instructions. The bead-beating step was performed using a homogenizer (FastPrep-24, MP Biomedicals) for 60 s at speed of 4 m/s.

### Microbiome analysis

Bacterial tag-encoded FLX-titanium amplicon pyrosequencing (bTEFAP) was performed as described previously [31] based upon the V4-V6 region (*E. coli* position 530 – 1100) of the 16S rRNA gene, with primers forward 530F: GTGCCAGCMGCNGCGG and reverse 1100R: GGGTTNCGNTCGTTR.

Raw sequence data were screened, trimmed, filtered, de-noised, and chimera-depleted with default settings using the QIIME pipeline version 1.6.0 (http://qiime.sourceforge.net) and with USEARCH using the OTU pipeline (www.drive5.com). Operational taxonomic units (OTUs) were defined as sequences with at least 97% similarity using QIIME. For classification of sequences on a genus level the naïve Bayesian classifier within the Ribosomal Database Project (RDP, v10.28) was used [31].

The obtained data were compiled to determine the relative proportions of bacteria for each individual sample. The subsequent analysis was performed on a randomly selected subset of 1,300 sequences per sample to account for unequal sequencing depth across samples. Alpha diversity and beta diversity measures were calculated and plotted using QIIME. To determine differences in microbiota composition between the animal groups, the analysis of similarities (ANOSIM) function in the statistical software package PRIMER 6 (PRIMER-E Ltd., Lutton, UK) was used on the unweighted Unifrac distances matrices. This analysis measures the phylogenetic distance among bacterial communities in a phylogenetic tree, and thereby provides a measure of similarity among microbial communities present in different biological samples. The linear discriminant analysis (LDA) effect size (LEfSe) method was used to represent taxonomic relevant age-related differences in foal fecal swabs [32].

### qPCR

To validate the pyrosequencing results, quantitative PCR (qPCR) assays were performed as described previously [33]. Briefly, EvaGreen-based reaction mixtures (total 10 µL) contained 5 µL of SsoFast™ EvaGreen® supermix (Biorad Laboratories), 2.2 µL of water, 0.4 µL of each primer (final concentration: 400 nM), and 2 µL of DNA (normalized to 5 ng/ul)). PCR conditions were 98°C for 2 min, and 40 cycles at 98°C 5 s, and 5 s at the optimized annealing temperature (Table 1). A melt curve analysis was performed for under the following conditions: beginning at 65°C, gradually increasing 0.5°C/5 s to 95°C with acquisition data every 5 s. The qPCR data was expressed as log amount of DNA (fg) for each particular bacterial group per 10 ng of isolated total DNA [34].

**Table 1:** Oligonucleotide primers/probes used for this study.

qPCR primers/probe	Sequence (5′- 3′)	Target	Annealing (°C)	Reference
CFB555f	CCGGAWTYATTGGGTTTAAAGGG	Bacteroidetes	60	56
CFB968r	GGTAAGGTTCCTCGCGTA			
Fuso-F	KGGGCTCAACMCMGTATTGCGT	Fusobacteria	51	26
Fuso-R	TCGCGTTAGCTTGGGCGCTG			
341-F	CCTACGGGAGGCAGCAGT	Universal Bacteria	59	57
518-R	ATTACCGCGGCTGCTGG			
EntF	CCCTTATTGTTAGTTGCCATCATT	*Enterococcus*	61	58
EntR	ACTCGTTGTACTTCCCATTGT			
EcolRT_F	GTTAATACCTTTGCTCATTGA	*E. coli*	55	59
EcolRT R	ACCAGGGTATCTAATCCTGTT			

### Data analysis

Pairwise comparisons between ages 2 days and 30 days were made at the levels of phylum, class, order, and family of bacteria for 2 outcomes: the observed percentage of sequences of bacteria at a given level, and the proportion of foals in which any amount of a given sequence for a given level was observed (i.e., the dichotomous outcome of whether or not a specific phylum [or class or order or family] was represented). The paired differences in percentages were compared using a Wilcoxon sign-rank test, and the paired proportions were compared using McNemar’s test. Because of the multiplicity of comparisons, P values at a given level (e.g., order) were adjusted using the method of Hochberg [35]. An adjusted P value <0.05 was considered significant for these analyses. Analyses were conducted using S-PLUS (Version 8.0; Insightful, Inc.) and R (Version 2.12.1; R Statistical Project). To assess the diversity of the GI microbiota, the Shannon-Weaver [36] and Chao 1 [37] diversity indices were calculated in QIIME.

## Results

### Sequence analysis

The 454-pyrosequencing pipeline yielded 499,419 quality sequences for the 42 samples analyzed. For technical reasons attributed to random error, 5 foals (2 foals from the control group without CTB, and 3 foals from the live *R. equi* group) did not generate sufficient sequences (cut-off value of 1,300 sequences) in at least 1 sample from 1 sampling time-point (either 2 or 30 days) by 454-pyrosequencing. Those foals were included in the descriptive analysis (Figures PCoA and rarefaction). For comparing age-related changes of the microbiome, however, the analysis was restricted to 37 foals with samples available from both collection time-points (2 and 30 days).

**Figure 1:**
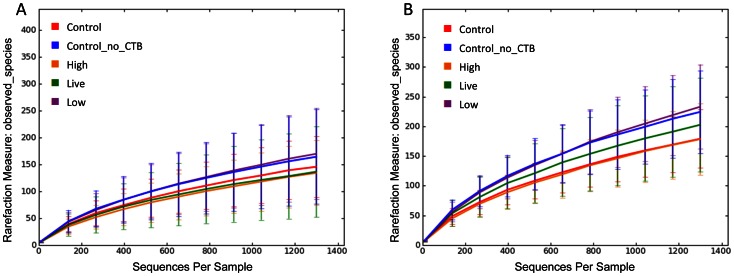
Rarefaction analysis of 16 S rRNA gene sequences obtained from fecal swabs from foals. Lines represent the average of each vaccination group at all ages (panel A) or at 30 days only (panel B), while the error bars represent the standard deviations. The analysis was performed on a randomly selected subset of 1,300 sequences per sample and included samples from 42 foals. Note that both the greatest and least number of species observed occurred among foals that received no enteral bacteria (live or inactivated), indicating an absence of evidence of treatment effect. Control  =  control plus CTB group; Control_no_CTO  =  control without CTB group; High  =  high-dose inactivated *R. equi* group; Live  =  live *R. equi* group; Low  =  low-dose inactivated *R. equi* group.

**Figure 2:**
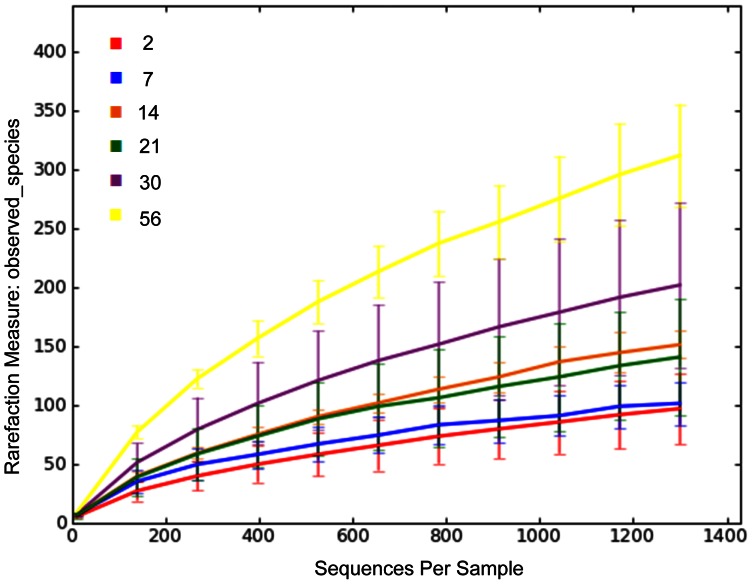
Rarefaction analysis of 16 S rRNA gene sequences obtained from fecal swabs from foals. Lines represent the average numbers obtained at each age (legend numbers refer to the age in days), while the error bars represent the standard deviations. The analysis was performed on a randomly selected subset of 1,300 sequences per sample and included samples from 42 foals. Note the progressive increase in observed species (representing microbial diversity) with sequential age. The numbers for the legend represent age (in days).

Across all vaccination groups and ages, sequences were classified into 18 phyla (Table 2 and 3). For the rarefaction curves of all vaccination groups (Figure 1A and 1B) and age groups (Figure 2), 1,300 sequences per sample yielded stable estimates of sample diversity.

**Table 2:** Median and range percentages of sequences represented in the fecal DNA of rectal swab samples from foals (Phylum, class, order, and family).

Microbial Phylum/Class/Order/Family				2-day-old foals (N = 37)	30-day-old foals(N = 37)	P*
Archaea.Euryarchaeota				0% (0%)	0% (0 to 0.6%)	**0.0048**
	Methanobacteria			0% (0%)	0% (0 to 0.3%)	**0.0280**
		Methanobacteriales		0% (0%)	0% (0 to 0.3%)	0.0924
			Methanobacteriacae	0% (0%)	0% (0 to 0.3%)	0.1932
	Methanomicrobia			0% (0%)	0% (0 to 0.6%)	0.1341
		Methanomicrobiales		0% (0%)	0% (0 to 0.6%)	0.4321
			Methanocorpusculaceae	0% (0%)	0% (0 to 0.6%)	0.9089
Bacteria.Acidobacteria				0% (0%)	0% (0 to 0.2%)	0.9515
	Acidobacteria			0% (0%)	0% (0 to 0.2%)	1.0000
		Acidobacteriales		0% (0%)	0% (0 to 0.2%)	1.0000
			Acidobacteriaceae	0% (0%)	0% (0 to 0.2%)	1.0000
Bacteria.Actinobacteria				0.2% (0 to 4.1%)	1.2% (0 to 4.3%)	**0.0048**
	Actinobacteria			0.2% (0 to 4.1%)	1.2% (0 to 4.3%)	**0.0280**
		Actinomycetales		0.1% (0 to 3.4%)	0.3% (0 to 2.8%)	0.3904
		Bifidobacteriales		0% (0 to 0.2%)	0% (0 to 0.2%)	1.0000
		Other		0% (0 to 0.7%)	0% (0 to 1.5%)	1.0000
	Coriobacteridae (subclass)			0% (0 to 1.5%)	0.2% (0 to 2.7%)	**<0.0001**
		Coriobacteriales		0% (0 to 1.5%)	0.2% (0 to 2.7%)	**0.0080**
	Rubrobacteridae (subclass)			0% (0 to 0.7%)	0% (0%)	0.9515
		Rubrobacterales		0% (0 to 0.7%)	0% (0%)	1.0000
		Other order		0% (0%)	0% (0 to 0.8%)	0.0578
			Other family	0% (0%)	0% (0 to 0.8%)	0.1190
Bacteria.Bacteroidetes				16.7% (0 to 85.5%)	40.6% (0.2 to 87.8%)	**0.0066**
	Bacteroidetes			16.7% (0 to 85.4%)	25.3% (0.1 to 80.5%)	0.5376
		Bacteroidales		16.7% (0 to 85.4%)	25.3% (0.2 to 80.5%)	0.9515
			Bacteroidieacae	16.7% (0 to 85.3%)	5.2% (0 to 53.3%)	1.0000
			Porphyromonadaceae	0% (0 to 9.0%)	0.4% (0 to 16.4%)	**0.0080**
			Prevotellaceae	0% (0 to 1.5%)	2.8% (0 to 63.1%)	**<0.0001**
			Rikenellaceae	0% (0 to 10.2%)	0% (0 to 5.8%)	0.2108
			Other	0% (0 to 0.2%)	4.0% (0 to 18.0%)	**<0.0001**
	Flavobacteria			0% (0 to 1.2%)	0% (0 to 2.3%)	0.8167
		Flavobacteriales		0% (0 to 1.2%)	0% (0 to 2.3%)	1.0000
			Flavobacteriaceae	0% (0 to 1.2%)	0% (0 to 2.3%)	1.0000
	Sphingobacteria			0% (0 to 0.2%)	0% (0 to 0.1%)	0.8167
		Sphingobacteriales		0% (0 to 0.2%)	0% (0 to 0.1%)	0.9515
			Crenotrichaceae	0% (0 to 0.1%)	0% (0%)	1.0000
			Flexibacteriaceae	0% (0%)	0% (0 to 0.1%)	1.0000
			Sphingobacteriaceae	0% (0 to 0.2%)	0% (0%)	1.0000
	Other class			0% (0 to 1.7%)	5.5% (0 to 48.1%)	**<0.0001**
		Other order		0% (0 to 1.7%)	5.5% (0 to 48.1%)	**<0.0001**
			Other family	0% (0 to 1.7%)	5.5% (0 to 48.1%)	**<0.0001**
Bacteria.Chlamydiae				0% (0%)	0% (0 to 30.1%)	**<0.0001**
	Chlamydiae			0% (0%)	0% (0 to 30.1%)	**<0.0001**
		Chlamydiales		0% (0%)	0% (0 to 30.1%)	**<0.0001**
			Chlamydiaceae	0% (0%)	0.1% (0 to 30.1%)	**<0.0001**
			Parachlamydiaceae	0% (0%)	0% (0 to 0.1%)	1.0000
Bacteria.Chloroflexi				0% (0%)	0% (0 to 0.2%)	**0.0441**
	Anaerolineae			0% (0%)	0% (0 to 0.2%)	0.2254
	Caldilineae			0% (0%)	0% (0 to 0.2%)	0.9515
		Caldilineales		0% (0%)	0% (0 to 0.2%)	1.0000
		Other		0% (0%)	0% (0 to 0.2%)	0.2635
			Other family	0% (0%)	0% (0 to 0.2%)	0.5355
Bacteria.Cyanobacteria				0% (0%)	0% (0 to 0.1%)	0.9515
	Cyanobacteria			0% (0%)	0% (0 to 0.1%)	1.0000
		Other order		0% (0%)	0% (0 to 0.1%)	1.0000
			Other family	0% (0%)	0% (0 to 0.1%)	1.0000
Bacteria.Deferribacteres				0% (0% to 0.2%)	0% (0 to 0.2%)	0.9515
	Deferribacteres			0% (0% to 0.2%)	0% (0 to 0.2%)	1.0000
		Deferribacterales		0% (0% to 0.2%)	0% (0 to 0.2%)	1.0000
			Deferribacteraceae	0% (0%)	0% (0%)	NP
			Incertae sedis 3	0% (0 to 0.2%)	0% (0 to 0.2%)	1.0000
Bacteria.Fibrobacteres				0% (0%)	0% (0 to 0.7%)	0.2104
	Fibrobacteres			0% (0% to 0.2%)	0% (0 to 0.7%)	0.4698
		Fibrobacterales		0% (0% to 0.2%)	0% (0 to 0.7%)	0.9515
			Fibrobacteraceae	0% (0% to 0.2%)	0% (0 to 0.7%)	1.0000
Bacteria.Firmicutes				40.4% (5.8 to 69.2%)	23.3% (4.4 to 95.2%)	0.9515
	Bacilli			4.8% (0.5 to 32.2%)	2.4% (0.1 to 78.8%)	0.2254
		Lactobacillales		4.8% (0.5 to 32.2%)	2.2% (0.1 to 69.8%)	0.6264
			Aerococcaceae	0% (0 to 1.6%)	0% (0 to 1.1%)	1.0000
			Carnobacteriaceae	0% (0 to 0.2%)	0% (0 to 0.1%)	1.0000
			Enterococcaceae	1.2% (0 to 14.5%)	0% (0 to 65.0%)	**0.0080**
			Lactobacillaceae	0% (0 to 7.9%)	0% (0 to 5.6%)	**0.0281**
			Leuconostocaceae	0% (0 to 0.2%)	0% (0%)	1.0000
			Streptococcaceae	2.1% (0 to 31.2%)	1.6% (0 to 20.8%)	1.0000
			Other	0.2% (0 to 1.7%)	0% (0 to 3.8%)	**0.0234**
		Bacillales		0% (0 to 0.3%)	0% (0 to 0.8%)	0.9515
			Paenibacillaceae	0% (0 to 0.2%)	0% (0 to 0.2%)	1.0000
			Staphylococcaceae	0% (0 to 2.5%)	0.1% (0 to 8.2%)	1.0000
			Bacillaceae	0% (0 to 0.3%)	0% (0 to 0.1%)	1.0000
			Incertae Sedis XI	0% (0 to 0.2%)	0% (0 to 0.1%)	1.0000
			Planococcaceae	0% (0%)	0% (0 to 0.2%)	1.0000
			Other	0% (0 to 0.2%)	0% (0 to 0.2%)	1.0000
		Other order		0% (0 to 0.1%)	0% (0 to 0.5%)	0.9515
			Other family	0% (0 to 0.1%)	0% (0 to 0.5%)	1.0000
	Clostridia			30.1% (3.4 to 64.5%)	18.8% (3.6 to 82.5%)	0.1314
		Clostridiales		30.1% (3.4 to 64.5%)	29.5% (3.4 to 64.5%)	0.9515
			Eubacteriaceae	0% (0 to 2.2%)	0% (0 to 1.2%)	0.3839
			Lachnospiraceae	3.7% (0 to 55.5%)	5.6% (0.7 to 76.7%)	1.0000
			Peptostreptococcaceae	3.4% (0 to 20.1%)	0% (0 to 12.4%)	**<0.0001**
			Ruminococcaceae	0.2% (0 to 4.6%)	1.5% (0.1 to 18.5%)	**<0.0001**
			Clostridiaceae	7.1% (0.1 to 45.2%)	5.4% (0 to 19.0%)	**0.0080**
			Incertae Sedis XI	0% (0 to 1.1%)	1.2% (0 to 14.0%)	**<0.0001**
			Incertae Sedis XIII	0% (0 to 0.8%)	0.1% (0 to 5.8%)	**0.0080**
			Peptococcaceae	0% (0%)	0% (0 to 2.5%)	**0.0438**
			Veillonellaceae	0% (0 to 8.3%)	0.9% (0 to 3.5%)	**<0.0001**
			Other	3.1% (0.1 to 13.7%)	2.0% (0 to 16.5%)	1.0000
		Other order		0% (0 to 0.4%)	0.2% (0 to 23.5%)	**0.0190**
			Other family	0% (0 to 0.4%)	0.2% (0 to 23.5%)	**0.0375**
	Erysipelotrichi			0.1% (0 to 1.0%)	0.1% (0 to 2%)	0.5376
		Erysipelotrichales		0.1% (0 to 1.0%)	0.1% (0 to 2%)	0.4844
			Erysipelotrichiaceae	0.1% (0 to 1.0%)	0.1% (0 to 2%)	1.0000
	Other class			0% (0 to 0.5%)	0.3% (0 to 6.5%)	**<0.0001**
		Other order		0% (0 to 0.5%)	0.3% (0 to 6.5%)	**<0.0001**
			Other family	0% (0 to 0.5%)	0.3% (0 to 6.5%)	**<0.0001**
Bacteria.Fusobacteria				0.8% (0 to 45.5%)	0.8% (0 to 42.5%)	0.9510
	Fusobacteria			0.8% (0 to 45.5%)	0.8% (0 to 42.2%)	1.0000
		Fusobacteriales		0.8% (0 to 45.5%)	0.8% (0 to 42.2%)	1.0000
			Fusobacteriaceae	0.4% (0 to 45.3%)	0.8% (0 to 42.2%)	1.0000
			Incertae sedis 11	0% (0 to 0.2%)	0% (0%)	1.0000
			Other	0% (0 to 16.1%)	0% (0 to 1.0%)	1.0000
Bacteria.Lentisphaerae				0% (0%)	0% (0%)	NP
	Lentisphaerae			0% (0%)	0% (0%)	NP
		Victivallales		0% (0%)	0% (0%)	NP
			Victivallaceae	0% (0%)	0% (0%)	NP
Bacteria. Other				0.2% (0 to 8.1%)	4.6% (0.2 to 68.5%)	**<0.0001**
	Bacteria. Other Class			0.2% (0 to 8.1%)	4.6% (0.2 to 68.5%)	**<0.0001**
		Other Order		0.2% (0 to 8.1%)	4.6% (0.2 to 68.5%)	**<0.0001**
			Other family	0.2% (0 to 8.1%)	4.6% (0.2 to 68.5%)	**<0.0001**
Bacteria.Planctomycetes				0% (0 to 0.1%)	0% (0 to 1.4%)	**0.0015**
	Planctomycetacia			0% (0 to 0.1%)	0% (0 to 1.4%)	**0.0322**
		Planctomycetales		0% (0 to 0.1%)	0% (0 to 1.4%)	**0.0047**
			Planctomycetaceae	0% (0 to 0.1%)	0% (0 to 1.4%)	**0.0080**
Bacteria.Proteobacteria				36.3% (0.5 to 85.8%)	2.7% (0 to 40.9%)	**<0.0001**
	Alphaproteobacteria			0% (0 to 0.3%)	0% (0 to 0.3%)	0.8167
		Caulobacterales		0% (0 to 0.2%)	0% (0)	0.9515
			Caulobacteriaceae	0% (0 to 0.2%)	0% (0)	1.0000
		Rhizobiales		0% (0 to 0.2%)	0% (0 to 0.2%)	0.9515
			Hyphomicrobiaceae	0% (0%)	0% (0 to 0.1%)	1.0000
			Methylobacteriaceae	0% (0 to 0.1%)	0% (0 to 0.1%)	1.0000
			Other	0% (0 to 0.2%)	0% (0 to 0.3%)	1.0000
		Rhodobacteriales		0% (0 to 0.2%)	0% (0 to 0.1%)	0.9515
			Rhodobacteriaceae	0% (0 to 0.2%)	0% (0 to 0.1%)	1.0000
		Rhodospirales		0% (0%)	0% (0%)	NP
			Other	0% (0%)	0% (0%)	NP
		Other order		0% (0%)	0% (0 to 0.1%)	0.9515
			Other family	0% (0%)	0% (0 to 0.1%)	1.0000
	Betaproteobacteria			0% (0 to 0.2%)	0% (0 to 0.5%)	0.4698
		Burkholderiales		0% (0 to 0.2%)	0% (0 to 0.5%)	0.9230
			Alcaligenaceae	0% (0 to 0.1%)	0% (0 to 0.1%)	1.0000
			Comamonadacea	0% (0 to 0.1%)	0% (0 to 0.1%)	1.0000
			Other	0% (0 to 0.1%)	0% (0 to 0.5%)	1.0000
		Other order		0% (0 to 0.1%)	0% (0 to 0.1%)	0.9515
			Other family	0% (0 to 0.1%)	0% (0 to 0.1%)	1.0000
	Deltaproteobacteria			0% (0 to 0.4%)	0% (0 to 1.1%)	**0.0084**
		Desulfovibrionales		0% (0 to 0.4%)	0% (0 to 0.5%)	**0.0385**
			Desulfovibrionaceae	0% (0 to 0.4%)	0% (0 to 0.5%)	0.1136
			Other	0% (0 to 0.1%)	0% (0 to 0.2%)	1.0000
		Myxococcales		0% (0%)	0% (0 to 1.1%)	0.9515
			Nannocystineae	0% (0%)	0% (0 to 1.0%)	1.0000
			Other	0% (0%)	0% (0 to 0.1%)	1.0000
	Epsilonproteobacteria			0% (0 to 0.1%)	0.3% (0 to 16.4%)	**<0.0001**
		Campylobacterales		0% (0 to 0.1%)	0.3% (0 to 16.4%)	**<0.0001**
			Campylobacteriaceae	0% (0 to 0.1%)	0.2% (0 to 4.7%)	**<0.0001**
			Helicobacteraceae	0% (%)	0% (0 to 15.9%)	**0.0080**
	Gamma proteobacteria			36.3% (0 to 85.8%)	0.5% (0 to 40.9%)	**<0.0001**
		Aeromonadales		0% (0 to 3.2%)	0% (0 to 4.2%)	0.9515
			Aeromonadaceae	0% (0 to 3.2%)	0% (0%)	0.3234
			Succinivibrionaceae	0% (0%)	0% (0 to 4.2%)	**0.0080**
		Enterobacteriales		36.2% (0 to 85.8%)	0.1% (0 to 39.8%)	**<0.0001**
			Enterobacteriaceae	36.2% (0 to 85.8%)	0.1% (0 to 39.8%)	**<0.0001**
		Legionellales		0% (0%)	0% (0 to 0.2%)	0.9515
			Coxiellaceae	0% (0%)	0% (0 to 0.1%)	1.0000
			Legionellaceae	0% (0%)	0% (0 to 0.2%)	1.0000
		Oceanospirillales		0% (0 to 0.1%)	0% (0%)	0.9515
			Halomonadaceae	0% (0 to 0.1%)	0% (0%)	1.0000
		Pasteurellales		0% (0 to 3.6%)	0% (0 to 1.2%)	0.9515
			Pasteurellaceae	0% (0 to 3.6%)	0% (0 to 1.2%)	1.0000
		Pseudomonadales		0% (0 to 1.5%)	0% (0 to 1.1%)	0.9515
			Moraxellaceae	0% (0 to 0.3%)	0% (0 to 0.7%)	1.0000
			Pseudomonadaceae	0% (0 to 1.2%)	0% (0 to 0.4%)	1.0000
		Xanthomonadales		0% (0%)	0% (0 to 0.1%)	0.9515
			Xanthomonadaceae	0% (0%)	0% (0 to 0.1%)	1.0000
		Other order		0% (0 to 0.5%)	0% (0%)	0.3278
			Other family	0% (0 to 0.5%)	0% (0%)	1.0000
	Other class			0% (0 to 0.2%)	0% (0 to 23.7%)	**0.0099**
		Other order		0% (0 to 0.2%)	0% (0 to 23.7%)	**0.0333**
			Other family	0% (0 to 0.2%)	0% (0 to 23.7%)	0.9089
Bacteria.Spirochaetes				0% (0%)	0% (0 to 2.1%)	**0.0100**
	Spirochaetes			0% (0%)	0% (0 to 2.1%)	**0.0375**
		Spirochaetales		0% (0%)	0% (0 to 2.1%)	**0.0360**
			Spirochaetaceae	0% (0%)	0% (0 to 2.1%)	0.0720
			Other	0% (0%)	0% (0 to 0.2%)	1.0000
Bacteria.TM7				0% (0%)	0% (0 to 1.8%)	**0.0048**
	TM7 genera incertae sedis			0% (0%)	0% (0 to 1.8%)	**0.0280**
		Other order		0% (0%)	0% (0 to 1.8%)	**0.0156**
			Other family	0% (0%)	0% (0 to 1.8%)	**0.0308**
Bacteria.Tenericutes				0% (0%)	0% (0 to 0.1%)	0.9515
	Mollicutes			0% (0%)	0% (0 to 0.1%)	1.0000
		Anaeroplasmatales		0% (0%)	0% (0 to 0.1%)	1.0000
			Anaeroplasmataceae	0% (0%)	0% (0 to 0.1%)	1.0000
Bacteria.Verrucomicrobia				0% (0 to 42.5%)	1.0% (0.4 to 48.7%)	**0.0015**
Verrucomicrobiae				0% (0 to 42.5%)	1.0% (0.4 to 48.7%)	**0.0322**
	Verrucomicrobiales			0% (0 to 42.5%)	1.0% (0.4 to 48.7%)	**0.0040**
		Other		0% (0%)	0% (0 to 0.2%)	1.0000
		Subdivision 5		0% (0 to 0.2%)	0.3% (0 to 25.4%)	0.0000
		Verrucomicrobiaceae		0% (0 to 42.5%)	0.6% (0 to 48.6%)	**0.0158**
		Xiphinematobacteriaceae		0% (0%)	0% (0 to 0.2%)	1.0000
Other Kingdom, Other phylum				0% (0 to 1.1%)	0% (0 to 0.3%)	0.9515
	Other class			0% (0 to 1.1%)	0% (0 to 0.3%)	1.0000
		Other order		0% (0 to 1.1%)	0% (0 to 0.3%)	1.0000
			Other family	0% (0 to 1.1%)	0% (0 to 0.3%)	1.0000

Fecal swab samples were collected from 37 Quarter Horse foals on days 2 and 30 of life. *P values represent the results of Wilcoxon sign-rank tests for paired differences, adjusted by the method of Hochberg. NP  =  Not Performed.

**Table 3:** Median and range proportion of foals with sequences detected in the fecal DNA of rectal swab samples (Phylum, class, order, and family).

Microbial Family				2-day-old foals (N = 37)	30-day-old foals (N = 37)	P*
Archaea.Euryarchaeota				0% (0/37)	35% (13/37)	0.0117
	Methanobacteria			0% (0/37)	24% (9/37)	0.0770
		Methanobacteriales		0% (0/37)	24% (9/37)	0.1925
			Methanobacteriacae	0% (0/37)	24% (9/37)	0.4851
	Methanomicrobia			0% (0/37)	16% (6/37)	0.3708
		Methanomicrobiales		0% (0/37)	16% (6/37)	0.9064
			Methanocorpusculaceae	0% (0/37)	16% (6/37)	1.0000
Bacteria.Acidobacteria				0% (0/37)	3% (1/37)	0.9999
	Acidobacteria			0% (0/37)	3% (1/37)	1.0000
		Acidobacteriales		0% (0/37)	3% (1/37)	1.0000
			Acidobacteriaceae	0% (0/37)	3% (1/37)	1.0000
Bacteria.Actinobacteria				73% (27/37)	97% (36/37)	0.1590
	Actinobacteria			73% (27/37)	97% (36/37)	0.1925
		Actinomycetales (order)		62% (23/37)	73% (27/37)	1.0000
		Bifidobacteriales (order)		3% (1/37)	8% (3/37)	1.0000
		Other		14% (5/37)	8% (3/37)	1.0000
	Coriobacteridae (subclass)			24% (9/37)	76% (28/37)	**0.0041**
		Coriobacteriales		24% (9/37)	76% (28/37)	**0.0221**
	Rubrobacteridae (subclass)			3% (1/37)	0% (0/37)	1.0000
		Rubrobacterales		3% (1/37)	0% (0/37)	1.0000
		Other order		0% (0/37)	27% (10/37)	0.1452
			Other family	0% (0/37)	27% (10/37)	0.2944
Bacteria.Bacteroidetes				92% (34/37)	100% (37/37)	0.5152
	Bacteroidetes			89% (33/37)	100% (37/37)	1.0000
		Bacteroidales		89% (33/37)	100% (37/37)	1.0000
			Bacteroidieacae	86% (32/37)	95% (35/37)	1.0000
			Porphyromonadaceae	30% (11/37)	89% (33/37)	**<0.0001**
			Prevotellaceae	8% (3/37)	95% (35/37)	**<0.0001**
			Rikenellaceae	11% (4/37)	49% (18/37)	0.1496
			Other	11% (4/37)	95% (35/37)	**<0.0001**
	Flavobacteria			16% (6/37)	11% (4/37)	1.0000
		Flavobacteriales		16% (6/37)	11% (4/37)	1.0000
			Flavobacteriaceae	16% (6/37)	11% (4/37)	1.0000
	Sphingobacteria			5% (2/37)	3% (1/37)	1.0000
		Sphingobacteriales		5% (2/37)	3% (1/37)	1.0000
			Crenotrichaceae	3% (1/37)	0% (0/37)	1.0000
			Flexibacteriaceae	0% (0/37)	3% (1/37)	1.0000
			Sphingobacteriaceae	3% (1/37)	0% (0/37)	1.0000
	Other class			27% (10/37)	95% (35/37)	**<0.0001**
		Other order		27% (10/37)	95% (35/37)	**<0.0001**
			Other family	27% (10/37)	95% (35/37)	**<0.0001**
Bacteria.Chlamydiae				0% (0/37)	51% (19/37)	**<0.0001**
	Chlamydiae			0% (0/37)	51% (19/37)	**<0.0001**
		Chlamydiales		0% (0%)	51% (19/37)	**<0.0001**
			Chlamydiaceae	0% (0%)	51% (19/37)	**<0.0001**
			Parachlamydiaceae	0% (0%)	3% (1/37)	1.0000
Bacteria.Chloroflexi				0% (0/37)	22% (8/37)	0.1463
	Anaerolineae			0% (0/37)	22% (8/37)	0.1173
	Caldilineae			0% (0/37)	3% (1/37)	1.0000
		Caldilineales		0% (0/37)	3% (1/37)	1.0000
		Other		0% (0/37)	19% (7/37)	0.7223
			Other family	0% (0/37)	19% (7/37)	1.0000
Bacteria.Cyanobacteria				0% (0/37)	3% (1/37)	0.9999
	Cyanobacteria			0% (0/37)	3% (1/37)	1.0000
		Other order		0% (0/37)	3% (1/37)	1.0000
			Other family	0% (0/37)	3% (1/37)	1.0000
Bacteria.Deferribacteres				3% (1/37)	5% (2/37)	0.9999
	Deferribacteres			3% (1/37)	5% (2/37)	1.0000
		Deferribacterales		3% (1/37)	5% (2/37)	1.0000
			Deferribacteraceae	0% (0%)	0% (0%)	NP
			Incertae sedis 3	3% (1/37)	5% (2/37)	1.0000
Bacteria.Fibrobacteres				0% (0/37)	14% (5/37)	0.5152
	Fibrobacteres			0% (0/37)	14% (5/37)	0.4698
		Fibrobacterales		0% (0/37)	14% (5/37)	0.9515
			Fibrobacteraceae	0% (0/37)	14% (5/37)	1.0000
Bacteria.Firmicutes				100% (37/37)	100% (37/37)	NP
	Bacilli			100% (37/37)	100% (37/37)	NP
		Lactobacillales		100% (37/37)	100% (37/37)	NP
			Aerococcaceae	27% (10/37)	14% (5/37)	1.0000
			Carnobacteriaceae	14% (5/37)	3% (1/37)	1.0000
			Enterococcaceae	95% (35/37)	41% (15/37)	**0.0078**
			Lactobacillaceae	27% (10/37)	70% (26/37)	0.2944
			Leuconostocaceae	8% (3/37)	0% (0/37)	1.0000
			Streptococcaceae	97% (36/37)	97%(36/37)	1.0000
			Other	78% (29/37)	22% (8/37)	**<0.0001**
		Bacillales		46% (17/37)	70% (26/37)	1.0000
			Paenibacillaceae	3% (1/37)	5% (2/37)	1.0000
			Staphylococcaceae	32% (12/37)	62% (23/37)	1.0000
			Bacillaceae	14% (5/37)	14% (5/37)	1.0000
			Incertae Sedis XI	11% (4/37)	3% (1/37)	1.0000
			Planococcaceae	3% (1/37)	11% (4/37)	1.0000
			Other	0% (0/37)	8% (3/37)	1.0000
		Other order		30% (11/37)	11% (4/37)	1.0000
			Other family	30% (11/37)	11% (4/37)	1.0000
	Clostridia			100% (37/37)	100% (37/37)	NP
		Clostridiales		100% (37/37)	100% (37/37)	NP
			Eubacteriaceae	5% (2/37)	35% (13/37)	0.6076
			Lachnospiraceae	89% (33/37)	100% (37/37)	1.0000
			Peptostreptococcaceae		43% (16/37)	**0.0150**
			Ruminococcaceae	70% (26/37)	100% (37/37)	0.1716
			Clostridiaceae	100% (37/37)	78% (29/37)	0.7980
			Incertae Sedis XI	11% (4/37)	78% (29/37)	**<0.0001**
			Incertae Sedis XIII	5% (2/37)	57% (21/37)	**0.0154**
			Peptococcaceae	0% (0/37)	32% (12/37)	0.1065
			Veillonellaceae	16% (6/37)	95% (35/37)	**<0.0001**
			Other	100% (37/37)	97% (36/37)	1.0000
		Other order		32% (12/37)	59% (22/37)	0.8136
			Other family	32% (12/37)	59% (22/37)	1.0000
	Erysipelotrichi			62% (23/37)	70% (26/37)	1.0000
		Erysipelotrichales		62% (23/37)	70% (26/37)	1.0000
			Erysipelotrichiaceae	62% (23/37)	70% (26/37)	1.0000
	Other class			49% (18/37)	86% (32/37)	**0.0242**
		Other order		49% (18/37)	86% (32/37)	0.0594
			Other family	49% (18/37)	86% (32/37)	1.0000
Bacteria.Fusobacteria				62% (23/37)	84% (31/37)	0.5152
	Fusobacteria			62% (23/37)	84% (31/37)	1.0000
		Fusobacteriales		62% (23/37)	84% (31/37)	1.0000
			Fusobacteriaceae	62% (23/37)	81% (30/37)	1.0000
			Incertae sedis 11	5% (2/37)	0% (0/37)	1.0000
			Other	32% (12/37)	43% (16/37)	1.0000
Bacteria.Lentisphaerae				0% (0/37)	0% (0/37)	NP
	Lentisphaerae			0% (0/37)	0% (0/37)	NP
		Victivallales		0% (0/37)	0% (0/37)	NP
			Victivallaceae	0% (0/37)	0% (0/37)	NP
Bacteria.Other				86% (32/37)	100% (37/37)	0.5152
	Bacteria. Other Class			86% (32/37)	100% (37/37)	1.0000
		Other Order		86% (32/37)	100% (37/37)	1.0000
			Other family	86% (32/37)	100% (37/37)	1.0000
Bacteria.Planctomycetes				3% (1/37)	43% (16/37)	**0.0045**
	Planctomycetacia			3% (1/37%)	43% (16/37)	**0.0377**
		Planctomycetales		3% (1/37)	43% (16/37)	**0.0120**
			Planctomycetaceae	3% (1/37)	43% (16/37)	**0.0003**
Bacteria.Proteobacteria				100% (37/37)	97% (36/37)	0.9999
	Alphaproteobacteria			11% (4/37)	14% (5/37)	1.0000
		Caulobacterales		3% (1/37)	0% (0/37)	1.0000
			Caulobacteriaceae	3% (1/37)	0% (0/37)	1.0000
		Rhizobiales		5% (2/37)	8% (3/37)	1.0000
			Hyphomicrobiaceae	0% (0/37)	3% (1/37)	1.0000
			Methylobacteriaceae	3% (1/37)	3% (1/37)	1.0000
			Other	3% (1/37)	3% (1/37)	1.0000
		Rhodobacteriales		5% (2/37)	3% (1/37)	1.0000
			Rhodobacteriaceae	5% (2/37)	3% (1/37)	1.0000
		Rhodospirales		0% (0%)	0% (0%)	NP
			Other	0% (0%)	0% (0%)	NP
		Other order		0% (0%)	3% (1/37)	1.0000
			Other family	0% (0%)	3% (1/37)	1.0000
	Betaproteobacteria			11% (4/37)	27% (10/37)	1.0000
		Burkholderiales		8% (3/37)	24% (9/37%)	1.0000
			Alcaligenaceae	3% (1/37)	3% (1/37)	1.0000
			Comamonadacea	3% (1/37)	8% (3/37)	1.0000
			Other	3% (1/37)	14% (5/37)	1.0000
		Other order		5% (2/37)	3% (1/37)	1.0000
			Other family	5% (2/37)	3% (1/37)	1.0000
	Deltaproteobacteria			5% (2/37)	49% (18/37)	**0.0104**
		Desulfovibrionales		5% (2/37)	46% (17/37)	**0.0377**
			Desulfovibrionaceae	5% (2/37)	43% (16/37)	0.1496
			Other	3% (1/37)	14% (5/37)	1.0000
		Myxococcales		0% (0/37)	3% (1/37)	1.0000
			Nannocystineae	0% (0/37)	3% (1/37)	1.0000
			Other	0% (0/37)	0% (0/37)	NP
	Epsilonproteobacteria			3% (1/37)	73% (27/37)	**<0.0001**
		Campylobacterales		3% (1/37)	73% (27/37)	**<0.0001**
			Campylobacteriaceae	3% (1/37)	65% (24/37)	**<0.0001**
			Helicobacteraceae	0% (0/37)	43% (16/37)	**0.0154**
	Gamma proteobacteria			97% (36/37)	89% (33/37)	1.0000
		Aeromonadales		22% (8/37)	41% (15/37)	1.0000
			Aeromonadaceae	22% (8/37)	0% (0/37)	0.7980
			Succinivibrionaceae	0% (0/37)	41% (15/37)	**0.0219**
		Enterobacteriales		95% (35/37)	62% (23/37)	0.0858
			Enterobacteriaceae	95% (35/37)	62% (23/37)	0.2145
		Legionellales		0% (0/37)	5% (2/37)	1.0000
			Coxiellaceae	0% (0/37)	3% (1/37)	1.0000
			Legionellaceae	0% (0/37)	3% (1/37)	1.0000
		Oceanospirillales		3% (1/37)	0% (0/37)	1.0000
			Halomonadaceae	3% (1/37)	0% (0/37)	1.0000
		Pasteurellales		32% (12/37)	38% (14/37)	1.0000
			Pasteurellaceae	32% (12/37)	38% (14/37)	1.0000
		Pseudomonadales		14% (5/37)	11% (4/37)	1.0000
			Moraxellaceae	14% (5/37)	11% (4/37)	1.0000
			Pseudomonadaceae	5% (2/37)	3% (1/37)	1.0000
		Xanthomonadales		0% (0/37)	3% (1/37)	1.0000
			Xanthomonadaceae	0% (0/37)	3% (1/37)	1.0000
		Other order		16% (6/37)	0% (0/37)	0.9064
			Other family	16% (6/37)	0% (0/37)	1.0000
	Other class			5% (2/37)	43% (16/37)	**0.0242**
		Other order		5% (2/37)	43% (16/37)	0.0594
			Other family	5% (2/37)	43% (16/37)	0.1496
Bacteria.Spirochaetes				0% (0/37)	30% (11/37)	**0.0312**
	Spirochaetes			0% (0/37)	30% (11/37)	**0.0439**
		Spirochaetales		0% (0/37)	30% (11/37)	0.0910
			Spirochaetaceae	0% (0/37)	30% (11/37)	0.1716
			Other	0% (0/37)	3% (1/37)	1.0000
Bacteria.TM7				0% (0/37)	35% (13/37)	**0.0117**
	TM7 genera incertae sedis			0% (0/37)	35% (13/37)	**0.0218**
		Other order		0% (0/37)	35% (13/37)	**0.0351**
			Other family	0% (0/37)	35% (13/37)	0.0648
Bacteria.Tenericutes				0% (0/37)	3% (1/37)	1.0000
	Mollicutes			0% (0/37)	3% (1/37)	1.0000
		Anaeroplasmatales		0% (0/37)	3% (1/37)	1.0000
			Anaeroplasmataceae	0% (0/37)	3% (1/37)	1.0000
Bacteria.Verrucomicrobia				24% (9/37)	89% (33/37)	**<0.0001**
Verrucomicrobiae				24% (9/37)	89% (33/37)	**<0.0001**
	Verrucomicrobiales			24% (9/37)	89% (33/37)	**<0.0001**
		Other		0% (0%)	5% (2/37)	1.0000
		Subdivision 5		8% (3/37)	76% (28/37)	**<0.0001**
		Verrucomicrobiaceae		16% (6/37)	78% (29/37)	**<0.0001**
		Xiphinematobacteriaceae		0% (0%)	3% (1/37)	1.0000
Other Kingdom, Other phylum				19% (7/37)	22% (8/37)	0.9999
	Other class			19% (7/37)	22% (8/37)	1.0000
		Other order		19% (7/37)	22% (8/37)	1.0000
			Other family	19% (7/37)	22% (8/37)	1.0000

Fecal swab samples collected from 37 Quarter Horse foals on days 2 and 30 of life. *P values represent the results of McNemar’s test for paired dichotomous data, adjusted by the method of Hochberg. NP  =  Not Performed.

### Microbial communities in control and vaccinated foals

**Figure 3:**
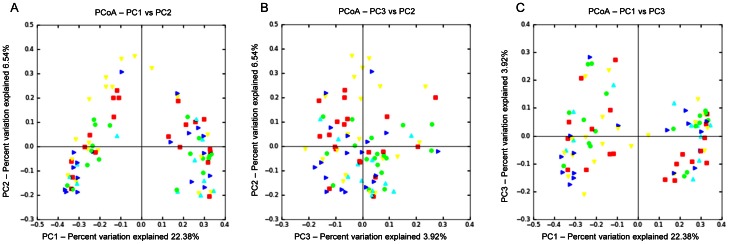
Principal coordinates analysis (PCoA) of unweighted UniFrac distances of 16 S rRNA genes. Analysis for 42 foals in groups control with CTB (red square), control without CTB (yellow triangle), low-dose inactivated *R. equi* (dark blue triangle), high-dose inactivated *R. equi* 2 (green dot), and live *R. equi* (light blue triangle) at 2 and 30 days of age (ANOSIM, P = 0.236). The 3 panels represent the comparison of the first 2 principal components (A), the second and third principal components (B), and the first and third principal components (C). The pattern in the panel A is attributable to effects of age (please see Figures 4 and 5).

**Figure 4:**
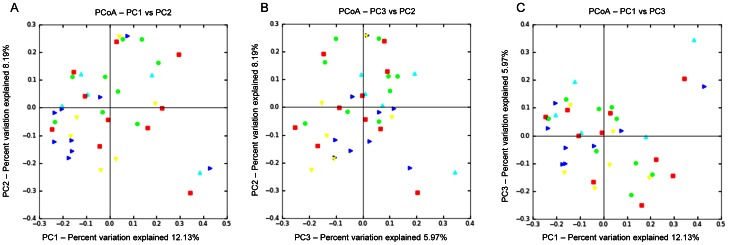
Principal coordinates analysis (PCoA) of unweighted UniFrac distances of 16 S rRNA genes. Analysis for 42 foals in groups control with CTB (red square), control without CTB (yellow triangle), low-dose inactivated *R. equi* (dark blue triangle), high-dose inactivated *R. equi* 2 (green dot), and live *R. equi* (light blue triangle) at 30 days of age only. Differences among groups were not significant (ANOSIM, P = 0.449). The 3 panels represent the comparison of the first 2 principal components (A), the second and third principal components (B), and the first and third principal components (C).

No differences in microbial composition were observed among animals from control, live and inactivated treatment/vaccination groups (Figures 1A, 1B, and 3). The rarefaction curves for the treatment groups revealed no clear pattern of greater number of observed species (i.e., diversity) among foals receiving either live or inactivated *R. equi*, or those foals in the 3 control groups that did not receive *R. equi* (Figure 1A). Because the samples at age 2 days were not affected by treatment (because treatment was administered after sample collection on day 2), we also performed analysis restricting data to samples collected at age 30 days (Figure 1B). Once again, there was no pattern of differences in the rarefaction curves among treatment groups receiving either live or inactivated *R. equi* or the control groups. Using PCoA (Figures 3 and 4), there was no qualitative evidence of differences among groups; the clustering observed in Figure 3 panel A was attributable to effects of age (please see next section). When considering only the data from foals at 30 days of age (because samples on day 2 were collected prior to treatment administration), the PCoA plots revealed no clustering by group and the ANOSIM test statistic for differences among groups was not significant (P = 0.494).

### Age-related changes in microbial communities in foals

**Figure 5:**
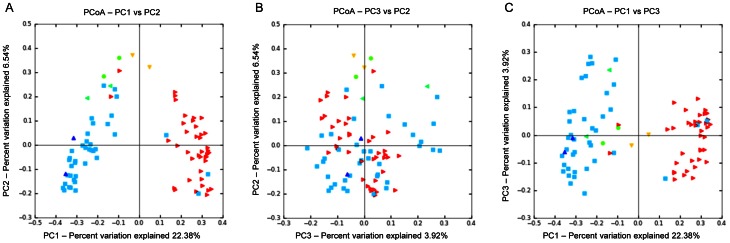
Principal coordinates analysis (PCoA) of unweighted UniFrac distances of 16 S rRNA genes. Analysis for 42 foals at 2 (red triangle), 7 days old (yellow triangle), 14 (green dot), 21 (green triangle), 30 (light blue square), and 56 days of age (dark blue triangle). The 3 panels represent the comparison of the first 2 principal components (A), the second and third principal components (B), and the first and third principal components (C). Strong effects of age can be seen in panels A and C, and differences among age groups were significant (ANOSIM, P  = 0.0010).

There were strong and significant differences in the fecal microbiome of foals associated with age. The rarefaction curves demonstrated a pattern of increasing number of species (diversity) with increasing age (Figure 2). These results should be interpreted with caution because there were only 2 foals for which data for ages other than 2 days and 30 days were available. The PCoA plots by age revealed an obvious separation of samples by age, attributable to differences between the time-points of days 2 and 30 (Figure 5); the ANOSIM test statistic for differences between day 2 and day 30 was significant (P = 0.0010).

Significant differences in the number of OTUs, the Shannon index, and the Chao1 metric were observed between the age groups (Table 4). The median number of OTUs for 2day-old foals (92 OTUs; range, 50 to 195 OTUs) was significantly (P<0.0001) lower than that for 30-day-old foals (201 OTUs; range, 94 to 318 OTUs). The Shannon Index for the foals studied also increased significantly (P<0.0001) from 2 days of life (median, 2.37; range, 1.24 to 3.97) to 30 days of life (median, 3.7; range, 1.90 to 4.80). Similarly, there was a significant (P<0.0001) age-related increase in Chao 1 values between 2-day-old foals (median, 206.54; range, 128.16 to 415.70) and 30-day-old foals (median, 362.38; range, 197.42 to 581.43).

**Table 4:** Summary of alpha diversity measures.

Index	2 day-old	30 day-old	P
Chao 1 (median, range)	206.54 (128.16 to 415.70)	362.38 (197.42 to 581.43)	**<0.0001**
OTUs (median, range)	92 (50 to 195)	201 (94 to 318)	**<0.0001**
Shannon H (median, range)	2.37 (1.24 to 3.97)	3.7 (1.90 to 4.80)	**<0.0001**

**Figure 6:**
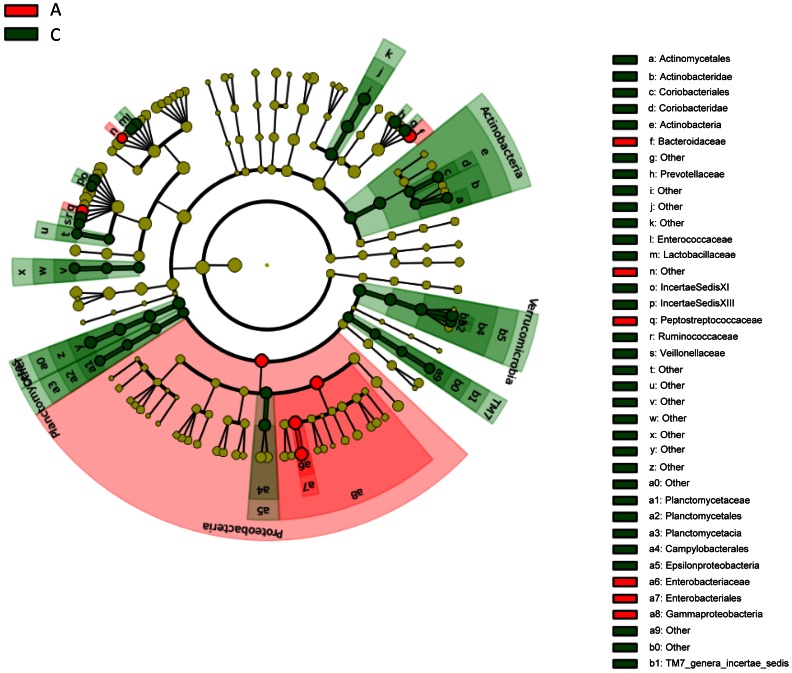
LEfSe results on foal microbiome. Rotary phylogenetic representation of the predominate microbial composition of fecal samples from foals at 2 days of age (A, red) and 30 days of age (C, green) [32].

Because of the apparent differences of the microbiota between age groups, we also compared the distribution of bacteria by phylum, class, order, and family between foals aged 2 days and 30 days. In total, 18 phyla were detected in fecal samples from foals (Table 2). Of those, Bacteroidetes (40.6%, day 30), Firmicutes (40.4%, day 2), and Proteobacteria (36.6%, day 2) had the highest percentages of sequences reported. Proteobacteria and Firmicutes were detected in all samples from 2-day-old foals, followed by Bacteroidetes (92%) and Actinobacteria (73%) (Table 3). Among 30-day-old foals, Bacteroidetes and Firmicutes were detected in all fecal samples, followed by Actinobacteria (97%) and Proteobacteria (97%), Verrucomicrobia (89%), and Fusobacteria (84%) (Table 3). The following phyla increased significantly with age (i.e, from 2 days to 30 days of age): Euryarchaeota, Actinobacteria, Bacteroidetes, Chlamydiae, Chloroflexi, Planctomycetes, Spirochaetes, TM7, and Verrucomicrobia. Proteobacteria was the only phylum that decreased significantly with age. Other classes, orders, and families also showed statistically significant age-related changes (Table 2 and Figure 6).

Within the phylum Proteobacteria, the class Gammaproteobacteria (P<0.0001) and the family Enterobacteriaceae (P<0.0001) decreased significantly with age. Other classes of Proteobacteria, such as Deltaproteobacteria (P = 0.0084) and Epsilonproteobacteria (P<0.0001) significantly increased with age (Table 2).

To confirm results of pyrosequencing, we also performed real-time quantitative PCR. Significant differences were observed in specific microbial communities between the 2 age groups based on qPCR analysis, with age-related decreases for *Escherichia coli* (P<0.0001) and for *Enterococcus* (P<0.0001). These data were consistent with genus-level results observed by pyrosequencing (Table 5) for *Enterococcus* (P = 0.0009) and for *Escherichia* (P<0.0001). We also found agreement for a lack of evidence of a significant difference between the pyrosequencing and the qPCR results for Bacteroidetes (P = 0.9519 by qPCR and P = 0.5376 by pyrosequencing) and Fusobacteria (P = 0.1051 on qPCR and P = 0.1000 on pyrosequencing).

**Table 5:** Results of qPCR analysis.

	Medians (min-max) log DNA (qPCR)		
	2 day-old	30 day-old	P*
Universal	13.2 (11.0 to 14.5)	12.3 (9.3 to 14.2)	**0.0108**
Bacteroidetes	11.4 (8.4 to 12.9)	11.2 (9.3 to 12.4)	0.9519
*Enterococcus*	7.9 (6.5 to 9.3)	5.7 (4.1 to 7.3)	**<0.0001**
*Escherichia*	8.2 (4. 3 to 8.9)	5.3 (2.8 to 6.5)	**<0.0001**
Fusobacteria	8.6 (6.0 to 10.4)	7.8 (6.4 to 9.7)	0.1051

Median (range) of log DNA. *P value for Wilcoxon rank-sum test comparing differences between ages day 30 and day 2, adjusted by the method of Hochberg.

## Discussion

In this study, our first objective was to evaluate changes in the microbiome of foals following vaccination with both live and inactivated *R. equi*. Although the number of CFUs administered were as high (for the live *R. equi* group) or higher than the number of CFU documented to protect foals against intrabronchial challenge with virulent *R. equi* (viz., 1×10^10^ CFU), no apparent differences in microbial communities were observed among vaccinated groups (Figures 1A and 3). Because all but 2 foals had samples collected only on days 2 and 30, and because fecal samples on day 2 were not influenced by treatment (because they were collected immediately prior to treatment), the effect of group also was examined among only samples collected at 30 days of age. Results restricted to 30 days of age also revealed no pattern distinguishing vaccinated and non-vaccinated foals (Figures 1B and 4). Thus, we failed to detect evidence of a significant effect of enteral administration of either live or inactivated *R. equi* on microbial populations in neonatal foals. These results are consistent with reports in which probiotics (administered at similar or higher numbers of CFUs) have failed to alter the intestinal/fecal microbiome [38]–[40]. Our results should be interpreted with caution because of the relatively small number of foals, particularly in the live *R. equi* group. For technical reasons attributed to random error, pyrosequencing failed for samples from 3 foals from the live *R. equi* group and 2 foals from the control group without CTB group; therefore, only 3 foals from the live *R. equi* group and 6 from the controls without CTB group were included in the analysis.

A significant difference between the fecal microbial populations between day 2 and day 30 of age was observed (Table 2 and 3; Figures 2, 5, and 6). For descriptive purposes, we included the results from the 2 foals from which we had data at other ages (these data were not included in the statistical analysis comparing ages). The resident intestinal or fecal microbiota has been described for neonates of other species, such as cats [41], [42], dogs [43], and humans [25]–[27], [44]. To the authors’ knowledge, this is the first report of age-related changes of the fecal microbiome in foals. Significant changes in the number of OTUs, the Shannon index, and the Chao1 metric were observed between the age groups (Table 4), showing clear evidence of strong diversification of bacterial populations between 2 and 30 days of age.

Firmicutes were detected in 100% of foals at both 2 and 30 days of age, with reported median sequences of 40% in 2-day-old foals decreasing (albeit not significantly) to 23% in 30-day-old foals. In 2 previous studies using fecal samples from adult horses, Firmicutes represented 44% [45] and 72% [46] of the bacteria. Within the Firmicutes, the family Enterococcaceae significantly decreased with age (P = 0.0080), which was likely attributable at least in part to decreases in the genus *Enterococcus* that were observed to decrease significantly by qPCR (P<0.0001) and by pyrosequencing (Table 5). Proteobacteria were detected in the feces of all 2-day-old foals and 97% of 30-day-old foals, a difference that was not significant; however, the median percentage of sequences decreased significantly (P<0.0001) between day 2 (median, 36.3%; range, 0.5 to 85.8%) and day 30 (median, 2.7%; range, 0 to 40.9%). In adult horses, Proteobacteria have been reported to represent 6% [45] and 12% [47] of fecal sequences. These results from adult horses are interesting in light of our findings, particularly our observation that the family Enterobacteriaceae decreased with age, a finding substantiated by our qPCR results with a significant decrease in the amount of *E. coli* (P<0.0001) between ages 2 and 30 days.

The sterile GI tract of newborn puppies and kittens is presumably colonized by bacteria present in the birth canal and from the environment [48], and human neonates appear to become colonized by these sources as well as through the intestinal microbiota of the mother [25], [49]. In humans, the initial microbes colonizing infants are facultative anaerobic bacteria, such as *E. coli* and *Streptococcus* spp. [49], which was also observed in 2-day old foals by the presence of Enterobacteriaceae (*E. coli*) and Streptococcaceae families (*Streptococcus* spp.). We observed a significant decrease in both these families by 30 days of age, suggesting that a similar phenomenon might happen in foals. In human beings, after the initial colonization by facultative anaerobic bacteria, colonization occurs by *Staphylococcus*-, *Enterococcus*-, and *Lactobacillus*-like species, and this change might contribute to generating an anaerobic environment [44]. The development during the first month of life in foals of an anaerobic environment is supported by the age-related increase in the detection of the phylum of Bacteroidetes (P = 0.0066), which is also a common constituent of the gut microbiota of dogs and cats [48]. However, we also observed a significant decrease in the Enterococcaceae family (P = 0.0080) and *Enterococcus* spp. by qPCR (P<0.0001), as well as the Lactobacillaceae family (P = 0.0281).

Our study has a number of important limitations. One limitation is the use of fecal swab samples for analysis, because feces might not be representative of other compartments of the gut. In humans, the composition of the mucosal-surface microbiota is distinct from that recovered in the feces [50]. The situation is probably similar in the horse, because of the complexity of the equine gastrointestinal tract. For example, the microbial population of adult horse fecal samples is likely to represent that of the right dorsal colon, but not that of the cecum [51].

A second limitation of our study is the small number of foals enrolled. Our sample size was limited both by financial considerations and the number of foals available to us during the study period. Because of the small sample size, we were only able to observe large changes in fecal microbial populations. Nevertheless, our results provide useful data for those exploring enteral vaccination of foals [14], [52]. It is worth noting that there were significant differences in immune responses that were detectable among these groups of foals despite the small sample size (data not shown). Also, we were able to detect significant age-related differences in the microbiome of foals, irrespective of the treatment groups.

Another limitation of our study is that we only characterized age-related changes at 2 ages during the first month of life. Although our data from 2 foals with more frequent sampling appears to demonstrate a progressive diversification of microbial flora with age (Figure 5), further studies using more foals with more frequent sampling times are needed to better characterize microbial diversification. Our focus on the first month of life was based on current understanding that vaccination of foals against *R. equi* will have to occur during early life [53].

In conclusion, no differences were observed in the fecal microbiome of foals following enteral vaccination with either live or inactivated *R. equi*. These results demonstrate that administration of the doses of bacteria used in this study does not likely cause an alteration of the fecal microbiome of foals. More notably, the results indicate significant age-related changes in the microbiome composition of foals during the first month of life.
